# The association of hyperglycaemia with prevalent tuberculosis: a population-based cross-sectional study

**DOI:** 10.1186/s12879-016-2066-1

**Published:** 2016-12-05

**Authors:** Sarah Lou Bailey, Helen Ayles, Nulda Beyers, Peter Godfrey-Faussett, Monde Muyoyeta, Elizabeth du Toit, John S. Yudkin, Sian Floyd

**Affiliations:** 1 LSHTM TB Centre and Department of Clinical Research, London School of Hygiene and Tropical Medicine, London, UK; 2 ZAMBART Project, Lusaka, Zambia; 3 Desmond Tutu TB Centre, Department of Paediatrics and Child Health, Stellenbosch University, Stellenbosch, South Africa; 4 University College London, London, UK; 5 LSHTM TB Centre and Department of Infectious Disease Epidemiology, London School of Hygiene and Tropical Medicine, London, UK

**Keywords:** Zambia, South Africa, Logistic regression

## Abstract

**Background:**

Systematic reviews suggest that the incidence of diagnosed tuberculosis is two- to- three times higher in those with diabetes mellitus than in those without. Few studies have previously reported the association between diabetes or hyperglycaemia and the *prevalence* of active tuberculosis and none in a population-based study with microbiologically-defined tuberculosis. Most have instead concentrated on cases of diagnosed tuberculosis that present to health facilities. We had the opportunity to measure glycaemia alongside prevalent tuberculosis. A focus on prevalent tuberculosis enables estimation of the contribution of hyperglycaemia to the population prevalence of tuberculosis.

**Methods:**

A population-based cross-sectional study was conducted among adults in 24 communities from Zambia and the Western Cape (WC) province of South Africa. Prevalent tuberculosis was defined by the presence of a respiratory sample that was culture positive for *M. tuberculosis*. Glycaemia was measured by random blood glucose (RBG) concentration. Association with prevalent tuberculosis was explored across the whole spectrum of glycaemia.

**Results:**

Among 27,800 Zambian and 11,367 Western Cape participants, 4,431 (15.9%) and 1,835 (16.1%) respectively had a RBG concentration ≥7.0 mmol/L, and 405 (1.5%) and 322 (2.8%) respectively had a RBG concentration ≥11.1 mmol/L. In Zambia, the prevalence of tuberculosis was 0 · 5% (142/27,395) among individuals with RBG concentration <11.1 mmol/L and also ≥11.1 mmol/L (2/405); corresponding figures for WC were 2 · 5% (272/11,045) and 4 · 0% (13/322). There was evidence for a positive linear association between hyperglycaemia and pulmonary prevalent tuberculosis. Taking a RBG cut-off 11.1 mmol/L, a combined analysis of data from Zambian and WC communities found evidence of association between hyperglycaemia and TB (adjusted odds ratio = 2 · 15, 95% CI [1 · 17–3 · 94]). The population attributable fraction of prevalent tuberculosis to hyperglycaemia for Zambia and WC combined was 0.99% (95% CI 0 · 12%–1.85%) for hyperglycaemia with a RBG cut-off of 11.1 mmol/L.

**Conclusions:**

This study demonstrates an association between hyperglycaemia and prevalent tuberculosis in a large population-based survey in Zambia and Western Cape. However, assuming causation, this association contributes little to the prevalence of TB in these populations.

## Background

The number of adults with diabetes mellitus globally is predicted to rise from 382 million in 2013 to 592 million in 2035 [1–6]. Of those in the world who currently have diabetes, four out of five live in a low or middle income country [6]. The burden of the anticipated rise in diabetes prevalence will fall largely to these low and middle income countries, the same countries that have some of the highest burdens of tuberculosis (TB) worldwide [4, 7].

Associations between diabetes and tuberculosis are increasingly recognised: systematic reviews and meta-analyses suggest that the incidence of active diagnosed tuberculosis is two- to- three times higher in those with diabetes compared to those without diabetes; [8–10] that diabetes increases the risk of death from diagnosed tuberculosis [11], and that diabetes may increase the risk of tuberculosis relapse [9, 11]. Studies from Africa have shown even stronger associations between diabetes and active diagnosed TB; a four-fold increase in diabetes prevalence was seen in TB patients in Dar es Salaam compared to the general population; [12] an odds ratio for TB of 8 · 33 was seen in the Congo comparing those with diabetes to those without diabetes [13]. As the prevalence of diabetes rises in locations with a high burden of tuberculosis, a deeper understanding of these associations is increasingly important.

We had the opportunity to measure glycaemia alongside tuberculosis in a population-based cross-sectional survey, which took place as part of the ZAMSTAR (Zambia South Africa TB and HIV Reduction Study) trial [14–16]. This trial was a 2 x 2 factorial community randomised trial to evaluate the impact of two complex interventions on the prevalence of TB in high HIV prevalence settings in Zambia and South Africa. The primary outcome for the study was the prevalence of tuberculosis after three years of intervention, measured through a cross-sectional survey of a random sample of adults from each community. We took this opportunity to also explore the association between hyperglycaemia and prevalent TB.

Both Zambia and South Africa have national TB control programmes, with a structured approach to TB diagnosis, management and control. Sputum smear microscopy is used for the diagnosis of pulmonary TB in Zambia. Few health care centres have access to culture or molecular tests. In South Africa Xpert MTB/RIF was introduced in 2011 for the diagnosis of pulmonary TB, though interruption to the supply of cartridges has been a challenge. Supply of TB medication rarely suffers interruption and is provided free of charge to patients in both countries. The situation for diabetes management is less favourable. In Zambia, point-of-care glucometers are most commonly used for diagnosis, though frequently glucometer strips are unavailable. Metformin and glibenclamide are widely available but access to alternative oral hypoglycaemics can be challenging. Insulin is available but can be difficult to access in remote areas. Storage of insulin is frequently problematic for patients due to lack of access to refrigeration. Unlike for TB, diabetes medication is not provided to patients for free.

Few studies have previously reported the association between diabetes or hyperglycaemia and *prevalent* tuberculosis in the general population, and to our knowledge none in a population-based study with microbiologically-defined tuberculosis. Most have concentrated on cases of tuberculosis that present to, and are diagnosed, at health facilities. Two historical studies did measure the prevalence of tuberculosis in the general population; one in Philadelphia, USA in 1946 [17], the other in Kristianstad, Sweden in 1954 [18]. Both identified pulmonary TB by chest radiograph, and identified diabetes through referrals from clinics and through medical records respectively. Rather than being based in a general population, other studies that have focused on tuberculosis prevalence have investigated the prevalence among patients with diabetes in a clinic setting or identified through medical records, using either no comparison group or clinic patients without diabetes as a comparison group [10, 19, 20]. Exploration of the association of hyperglycaemia with prevalent tuberculosis in a population-based study allows for estimation of the contribution of hyperglycaemia to the population prevalence of tuberculosis.

The aims of this study are therefore to determine the association between hyperglycaemia and prevalent tuberculosis and to estimate the population attributable fractions of prevalent tuberculosis to hyperglycaemia, assuming causation, within our study communities in Zambia and the Western Cape region of South Africa.

## Methods

This population-based cross-sectional study was nested within a 2 x 2 factorial cluster-randomised trial (the ZAMSTAR study [14–16]) and undertaken between January and December 2010 in 24 study communities: 16 from 5 provinces in Zambia and 8 from the Western Cape province of South Africa. Within each community, a two-stage cluster sampling design was used to recruit participants. Exclusion criteria were age <18 years, inability to give informed consent due to disability/incapacitation, refusal to submit a respiratory sample and any persons living in institutional settings.

Each participant was required to give written informed consent. Individuals and household heads were interviewed in their homes using structured questionnaires. Each participant was requested to produce a spot respiratory sample for tuberculosis culture. Finger prick capillary blood was taken for HIV testing and random blood glucose (RBG) measurement, with pre- and post-test counselling for HIV tests. RBG concentration was measured using an Optium Xceed point-of-care glucometer. All individuals identified to have abnormal blood glucose or to be HIV positive were referred to existing local health facilities for appropriate management.

Data were electronically entered directly onto personal digital assistants by field staff at the time of data collection, using pre-programmed questionnaires and result sheets. All information was downloaded daily into a SQL (structured query language) database and later exported into Stata.

All procedures for sputum sample collection and culture were identical in all study sites. Research staff in all sites were trained to instruct participants on adequate expectoration to achieve a lower rather than upper airways sample. Samples were collected daily from field sites and delivered to the laboratory in each study community. A standard liquid culture technique was used to isolate *Mycobacterium tuberculosis* (MGIT, Becton Dickinson). Growth detected by culture was identified using an immunochromatographic assay (Capilia TB), and all Capilia TB assay positive cultures were confirmed by 16S ribosomal RNA sequencing. Detailed methods are described by Ayles et al. in the final report of the ZAMSTAR trial [16].

The Optium Xceed glucometer uses a whole blood capillary sample but is calibrated to report the plasma equivalent result. The results presented here are therefore the plasma equivalent glucose concentrations. This device was chosen because of its documented accuracy in multiple independent studies combined with its availability in Zambia and South Africa. It was found to be one of the most superior glucometers in all published accuracy studies, with between 84% and 100% of the results from the finger prick capillary specimen being within the recommended limits compared to reference plasma estimation on laboratory analysers [21–27]. When inaccuracy was seen, this was mostly for low rather than high glucose concentrations [24, 25, 27]. All research staff were trained on the use of this particular glucometer and were required to undergo proficiency testing. Standardised control solution was used for performance checks on test strips and meters.

Ethics approval was granted from the London School of Hygiene and Tropical Medicine Ethics Committee, the University of Stellenbosch Human Research Ethics Committee and the University of Zambia Biomedical Research Ethics Committee.

### Definitions

- Hyperglycaemia, the exposure of interest for this study, is initially examined with RBG concentration as an ordered categorical variable. We then use sequential RBG cut-offs – 7.0 mmol/L, 7.8 mmol/L, 9.0 mmol/L and 11.1 mmol/L – to explore increasing levels of hyperglycaemia. We based our cut-off levels on the current World Health Organisation guidelines for diabetes diagnosis and monitoring [28], though this was only to allow for exploration of increasing levels of glycaemia and not intended to be indicative of diabetes diagnoses.
- Prevalent pulmonary tuberculosis, the outcome of interest for this study, is defined by the presence of a respiratory sample that is culture positive for *Mycobacterium tuberculosis*.
- HIV status is defined by a combination of blood sampling plus self-report for those with missing biological data.

### Analysis strategy

Principal components analysis was used to create a measure of household socio-economic position separately for each country. Unadjusted and adjusted odds ratios of the association between hyperglycaemia and prevalent tuberculosis were estimated using logistic regression analysis, accounting for within-cluster correlation resulting from the sampling design. Analyses were performed separately for Zambian and South African data due to heterogeneity between the two distinct locations. This enabled us to control for confounding differently for each location, which was necessary because of the big differences between the two settings. Fixed-effects meta-analysis of adjusted odds ratios from each country was then performed, with weights according to the inverse variance method to give overall odds ratios of the association between hyperglycaemia and prevalent tuberculosis. Population attributable fractions (PAFs) of prevalent tuberculosis to hyperglycaemia and HIV were calculated separately for each country and for combined estimates. These were calculated using the formula *PAF* = ∑*p*_*k*_ ' (*θ*_*k*_ − 1)/*θ*_*k*_ where p’ is the proportion of cases exposed in the study population at exposure level k and θ is the adjusted odds ratio. Given that θ and p’ were estimated from the same data, the 95% confidence intervals could be calculated using the following error factor for (1-PAF):

$$ Error\  factor= \exp \left\{1.96\times \sqrt{\frac{n_1{p^{\hbox{'}}}^2V+2{p}^{\hbox{'}}\left(\theta -1\right)+{p}^{\hbox{'}}\left(1-{p}^{\hbox{'}}\right){\left(\theta -1\right)}^2}{n_1{\left[\theta \left(1-{p}^{\hbox{'}}\right)+{p}^{\hbox{'}}\right]}^2}}\right\} $$

where n_1_ is the total number of cases observed and V the variance of the adjusted log odds ratio (the standard error of the log odds ratio squared). Evidence for effect modification by gender and HIV was explored. All data analyses were performed using Stata 13.

## Results

**Fig. 1:**
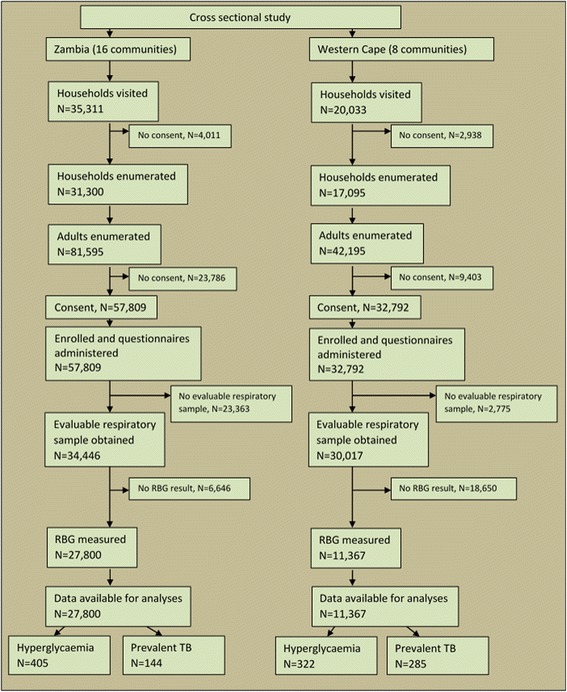
Number and flow of participants and cases in this cross sectional study in Zambia and the Western Cape of South Africa

The cross-sectional survey enrolled 57,809 (70 · 8% of eligible) participants from 31,300 (88 · 6% of eligible) households in Zambia and 32,792 (77 · 7% of eligible) participants from 17,095 (85 · 3% of eligible) households in Western Cape (Fig. 1). Evaluable sputum samples and complete RBG results were obtained for 27,800 (48 · 1% of enrolled) participants in Zambia and 11,367 (34 · 7% of enrolled) participants in Western Cape. Data from these participants were analysed. Comparison of individuals with evaluable sputum samples with those with non-evaluable samples in Zambia showed them to be much the same (data presented as supplementary material to the ZAMSTAR trial publication) [16].

Among Zambian and Western Cape participants, 15.9% and 16.1% respectively had a RBG concentration ≥7.0 mmol/L, and 1.5% and 2.8% respectively had a RBG concentration ≥11.1 mmol/L. The prevalence of tuberculosis was approximately 500 per 100,000 (0 · 5%) among Zambian participants and approximately 2,500 per 100,000 (2 · 5%) among Western Cape participants. Tuberculosis prevalence stratified by glycaemia and baseline characteristics is shown in Tables 1 and 2.

**Table 1:** Logistic regression estimates of the unadjusted and adjusted odds ratios of prevalent tuberculosis in the Zambian study sites

Characteristic		Total number (%)	Number (%) with prevalent TB	Unadjusted OR (95% CI)	*P*-value^#^	Adjusted^a^ OR (95% CI)	*P*-value^#^
Overall		27,800 (100)	144 (0 · 5)	-	-	-	-
Random blood glucose concentration (mmol/L)	<5.6	14,741 (53.0)	67 (0.5)	1	0.029 (test for linear trend *p* = 0.017; test for departure from trend *p* = 0.164)	1	0.011 (test for linear trend *p* = 0.006; test for departure from trend *p* = 0.141)
	5.6–6.9	8,628 (31.0)	42 (0.5)	1.06 (0.72–1.56)		1.13 (0.75–1.71)	
	7.0–8.9	3,447 (12.4)	24 (0.7)	1.51 (0.94–2.41)		1.6 (0.96–2.66)	
	9.0–11.0	579 (2.1)	9 (1.6)	3.44 (1.7–6.96)		4.31 (2.07–8.97)	
	>11.0	405 (1.5)	2 (0.5)	1.06 (0.26–4.35)		0.97 (0.13–7.14)	
Age (years)	18–24	9,733 (35 · 4)	37 (0 · 4)	1	0 · 004	1	0 · 390
	25–29	4,665 (17 · 0)	32 (0 · 7)	1 · 79 (1 · 11–2 · 88)		1.27 (0.75–2.16)	
	30–34	3,438 (12 · 5)	28 (0 · 8)	2 · 15 (1 · 31–3 · 52)		1.42 (0.83–2.46)	
	35–39	2,381 (8 · 7)	16 (0 · 7)	1 · 79 (0 · 99–3 · 24)		1.09 (0.57–2.09)	
	40–49	3,173 (11 · 5)	20 (0 · 6)	1 · 67 (0 · 97–2 · 89)		1.04 (0.56–1.92)	
	50–59	2,113 (7 · 7)	7 (0 · 3)	0 · 87 (0 · 39–1 · 96)		0.74 (0.32–1.72)	
	60+	1,998 (7 · 3)	4 (0 · 2)	0 · 55 (0 · 20–1 · 55)		0.49 (0.16–1.45)	
Sex	Male	9,265 (33 · 3)	64 (0 · 7)	1	0 · 003	1	0 · 008
	Female	18,535 (66 · 7)	80 (0 · 4)	0 · 60 (0 · 43–0 · 84)		0.59 (0.41–0.87)	
HIV status	Negative	21,103 (82 · 5)	72 (0 · 3)	1	<0 · 001	1	<0 · 001
	Positive	4,465 (17 · 5)	66 (1 · 5)	4 · 22 (3 · 00–5 · 92)		3.57 (2.44–5.23)	
Body Mass Index (weight(kg)/height^2^(m))	Healthy weight (18.5–24.9)	16,497 (64.2)	95 (0.6)	1	<0.001	1	<0.001
	Underweight (<18.5)	2,604 (10.1)	27 (1.0)	1.87 (1.22–2.89)		1.70 (1.09–2.65)	
	Overweight (25–29.9)	4,402 (17.1)	13 (0.3)	0.49 (0.27–0.88)		0.56 (0.3–1.05)	
	Obese (≥30)	2,214 (8.6)	1 (0.1)	0.07 (0.01–0.53)		0.10 (0.01–0.76)	
Region	Rural, low ARTI	7,712 (27 · 7)	24 (0 · 3)	1	0 · 012	1	0 · 002
	Urban (non-Lusaka), low ARTI	6,033 (21 · 7)	34 (0 · 6)	1 · 83 (1 · 02–3 · 29)		2.13 (1.12–4.04)	
	Urban (non-Lusaka), high ARTI	5,158 (18 · 6)	22 (0 · 4)	1 · 39 (0 · 74–2 · 63)		1.59 (0.81–3.14)	
	Lusaka, high ARTI	8,897 (32 · 0)	64 (0 · 7)	2 · 34 (1 · 37–3 · 98)		2.88 (1.62–5.11)	

*TB* tuberculosis, *OR* odds ratio, *CI* confidence interval, *ARTI* annual risk of infection; All analyses accounted for the two-stage clustered sampling design through the use of a logistic regression model with random effects for enumeration area and inclusion of region as a fixed effect, with each region including 4 communities; ^#^Likelihood ratio tests; ^a^23,414 participants included in analysis (299 missing data for age, 2,083 for BMI, 2,232 for HIV status), adjusted for variables shown plus household socioeconomic position and education

**Table 2:** Logistic regression estimates of the unadjusted and adjusted odds ratios of prevalent tuberculosis in the Western Cape study sites

Characteristic		Total number (%)	Number (%) with prevalent TB	Unadjusted OR (95% CI)	*P*-value^#^	Adjusted^a^ OR (95% CI)	*P*-value^#^
Overall		11,367 (100)	285 (2 · 5)	-	-	-	-
Random blood glucose concentration (mmol/L)	<5.6	6,068 (53.4)	146 (2.4)	1	0.149 (test for linear trend *p* = 0.328; test for departure from trend *p* = 0.121)	1	0.059 (test for linear trend *p* = 0.098; test for departure from trend *p* = 0.096)
	5.6–6.9	3,464 (30.5)	89 (2.6)	1.06 (0.81–1.39)		1.14 (0.85–1.53)	
	7.0–8.9	1,269 (11.2)	35 (2.8)	1.14 (0.78–1.66)		1.15 (0.76–1.76)	
	9.0–11.0	244 (2.2)	2 (0.8)	0.34 (0.08–1.40)		0.42 (0.10–1.76)	
	>11.0	322 (2.8)	13 (4.0)	1.71 (0.95–3.06)		2.49 (1.29–4.79)	
Age (years)	18–24	2,953 (26 · 0)	59 (2 · 0)	1	0 · 059	1	0 · 014
	25–29	1,650 (14 · 5)	40 (2 · 4)	1 · 19 (0 · 79–1 · 79)		1.25 (0.80–1.95)	
	30–34	1,325 (11 · 7)	24 (1 · 8)	0 · 89 (0 · 55–1 · 45)		0.81 (0.47–1.39)	
	35–39	1,175 (10 · 3)	39 (3 · 3)	1 · 67 (1 · 11–2 · 52)		1.70 (1.07–2.69)	
	40–49	1,902 (16 · 7)	52 (2 · 7)	1 · 36 (0 · 93–1 · 99)		1.34 (0.87–2.08)	
	50–59	1,302 (11 · 5)	42 (3 · 2)	1 · 63 (1 · 09–2 · 44)		2.11 (1.31–3.39)	
	60+	1,052 (9 · 3)	29 (2 · 8)	1 · 39 (0 · 88–2 · 20)		1.72 (0.96–3.06)	
Sex	Male	3,743 (32 · 9)	116 (3 · 1)	1	0 · 006	1	0 · 841
	Female	7,624 (67 · 1)	169 (2 · 2)	0 · 71 (0 · 56–0 · 90)		0.97 (0.73–1.29)	
HIV status	Negative	8,768 (82 · 3)	164 (1 · 9)	1	<0 · 001	1	<0 · 001
	Positive	1,889 (17 · 7)	97 (5 · 1)	2 · 83 (2 · 18–3 · 66)		2.96 (2.23–3.93)	
Body Mass Index (weight(kg)/height^2^(m))	Healthy weight (18.5–24.9)	4,302 (39.0)	132 (3.1)	1	<0.001	1	<0.001
	Underweight (<18.5)	605 (5.5)	53 (8.8)	2.98 (2.13–4.17)		3.04 (2.14–4.34)	
	Overweight (25–29.9)	2,609 (23.7)	56 (2.2)	0.69 (0.50–0.95)		0.58 (0.41–0.83)	
	Obese (≥30)	3,510 (31.8)	34 (1.0)	0.31 (0.21–0.45)		0.25 (0.16–0.39)	
Community	SA1	1,131 (10 · 0)	29 (2 · 6)	1	0 · 308	1	0 · 351
	SA2	2,193 (19 · 3)	49 (2 · 2)	0 · 84 (0 · 49–1 · 42)		0.85 (0.47–1.55)	
	SA3 (rural)	190 (1 · 7)	3 (1 · 6)	0 · 61 (0 · 18–2 · 15)		0.51 (0.11–2.39)	
	SA4	1,618 (14 · 2)	57 (3 · 5)	1 · 33 (0 · 79–2 · 25)		1.49 (0.82–2.73)	
	SA5	1,164 (10 · 2)	20 (1 · 7)	0 · 64 (0 · 34–1 · 22)		0.71 (0.33–1.51)	
	SA6 (rural)	2,489 (21 · 9)	65 (2 · 6)	1 · 01 (0 · 61–1 · 67)		1.01 (0.57–1.78)	
	SA7	1,557 (13 · 7)	40 (2 · 6)	1 · 02 (0 · 59–1 · 75)		1.05 (0.55–1.99)	
	SA8	1,025 (9 · 0)	22 (2 · 2)	0 · 83 (0 · 45–1 · 52)		0.86 (0.43–1.72)	

*TB* tuberculosis, *OR* odds ratio, *CI* confidence interval; All analyses accounted for the two-stage clustered sampling design through use of a logistic regression model with random effects for enumeration area and inclusion of community as a fixed effect; ^#^Likelihood ratio tests; ^a^10,336 participants included in analysis (8 missing data for age; 341 for BMI; 710 for HIV status), adjusted for variables shown plus household socioeconomic position and education

Among individuals included in the analysis, HIV status was determined by blood sampling for 23,067 (90 · 2%) participants and by self-reported status for 2,501 (9 · 8%) participants in Zambia. Corresponding values for Western Cape participants are 10,106 (94 · 8%) and 551 (5 · 2%). Among participants with RBG results, 2,232 participants in Zambia and 710 in Western Cape had missing data for both blood sampling and self-reported HIV status.

Among Zambian participants, as RBG concentration increased, the unadjusted and adjusted odds of prevalent TB initially increased compared to the baseline RBG concentration <5.6 mmol/L, peaking at RBG concentration 9.0–11.0 mmol/L (adjusted OR 4.31, 95% CI [2.07–8.97]). Although the odds of prevalent TB did not continue to increase with increasing RBG concentration beyond this, the number of individuals with a RBG concentration ≥11.1 mmol/L was low and the confidence interval was wide so there was still strong evidence of a linear association between RBG concentration and TB prevalence (*p* = 0.006) after adjusting for age, sex, household socio-economic position, education, body mass index, HIV status and geographical location (Table 1).

On multivariable analysis there was weak evidence of a linear association between glucose concentration and TB prevalence among Western Cape participants (p = 0.06). In this location the adjusted odds of prevalent TB compared to the baseline was greatest for individuals with a RBG concentration ≥11.1 mmol/L (OR 2.49, 95% CI [1.29–4.79], Table 2).

Unadjusted and adjusted odds ratios for prevalent TB using sequential RBG cut-offs to give increasing levels of hyperglycaemia are shown in Table 3. The findings from Zambia and Western Cape, combined with fixed-effects meta-analyses, showed increasing adjusted odds of prevalent tuberculosis for increasing cut-off levels of hyperglycaemia, though the increase was small; and, across successive cut-off levels, confidence intervals overlapped (Table 4). There was no evidence that the adjusted odds ratios differed between Zambia and Western Cape (Table 4).

**Table 3:** Logistic regression estimates of prevalent tuberculosis and population attributable fractions of prevalent tuberculosis to hyperglycaemia for sequential random blood glucose concentration cut-offs

Random blood glucose concentration (mmol/L)	Total number (%)	Number (%) with prevalent TB	Unadjusted OR (95% CI)	*P*-value^#^	Adjusted OR (95% CI)^a^	*P*-value^#^	PAF (95% CI) of prevalent TB to hyperglycaemia (%)
ZAMBIA							
<7.0	23,369 (84.1)	109 (0.5)	1	0.012	1	0.007	7.16 (2.51–11.59)
≥7.0	4,431 (15.9)	35 (0.8)	1.68 (1.14–2.46)		1.82 (1.20–2.75)		
<7.8	25,444 (91.5)	123 (0.5)	1	0.017	1	0.018	4.12 (0.90–7.24)
≥7.8	2,356 (8.5)	21 (0.9)	1.84 (1.15–2.93)		1.94 (1.16–3.25)		
<9.0	26,816 (96.5)	133 (0.5)	1	0.022	1	0.007	2.28 (0.12–4.38)
≥9.0	984 (3.5)	11 (1.1)	2.25 (1.21–4.19)		2.86 (1.46–5.60)		
<11.1	27,395 (98.5)	142 (0.5)	1	0.921	1	0.818	0.00 (0.00–3.21)
≥11.1	405 (1.5)	2 (0.5)	0.93 (0.23–3.79)		0.80 (0.11–5.85)		
WESTERN CAPE							
<7.0	9,532 (83.9)	235 (2.5)	1	0.525	1	0.386	2.34 (0.00–7.08)
≥7.0	1,835 (16.1)	50 (2.7)	1.11 (0.81–1.51)		1.17 (0.82–1.66)		
<7.8	10,320 (90.8)	255 (2.5)	1	0.417	1	0.383	1.66 (0.00–4.96)
≥7.8	1,047 (9.2)	30 (2.9)	1.18 (0.80–1.73)		1.22 (0.79–1.89)		
<9.0	10,801 (95.0)	270 (2.5)	1	0.787	1	0.308	1.35 (0.00–3.55)
≥9.0	566 (5.0)	15 (2.7)	1.08 (0.63–1.83)		1.37 (0.77–2.44)		
<11.1	11,045 (97.2)	272 (2.5)	1	0.099	1	0.015	1.64 (0.28–2.99)
≥11.1	322 (2.8)	13 (4.0)	1.67 (0.94–2.96)		2.38 (1.26–4.50)		

*TB* tuberculosis, *OR* odds ratio, *CI* confidence interval, *PAF* population attributable fraction; All analyses accounted for the two-stage clustered sampling design through the use of a logistic regression model with random effects for enumeration area and inclusion of region or community as a fixed effect; Negative PAFs were given a value of zero; ^#^Likelihood ratio tests; ^a^Adjusted for age, sex, HIV status, body mass index, household socioeconomic position and education

**Table 4:** Combined adjusted odds ratios of prevalent tuberculosis for Zambia and Western Cape and associated population attributable fractions of prevalent tuberculosis to hyperglycaemia for sequential random blood glucose concentration cut-offs

Random blood glucose concentration cut-off (mmol/L)	Combined adjusted OR*	*P*-value^#^	I^2^ *p*-value	PAF (95% CI) of prevalent TB to hyperglycaemia (%)
7.0	1.40 (1.07–1.84)	0.013	0.112	4.57 (1.27–7.77)
7.8	1.48 (1.06–2.07)	0.020	0.176	2.82 (0.64–4.95)
9.0	1.87 (1.21–2.90)	0.005	0.104	1.84 (0.56–3.11)
11.1	2.15 (1.17–3.94)	0.013	0.306	0.99 (0.12–1.85)

*OR* odds ratio, *CI* confidence interval, *PAF* population attributable fraction; ORs combined through fixed-effects meta-analysis; ^#^Likelihood ratio tests; All analyses accounted for the two-stage clustered sampling design through the use of a logistic regression model with random effects for enumeration area and inclusion of region or community as a fixed effect; Negative PAFs were given a value of zero; *Adjusted for age, sex, HIV status, body mass index, household socioeconomic position and education

The evidence for association between hyperglycaemia and prevalent tuberculosis strengthened from univariable to multivariable analyses in both Zambia and the Western Cape communities. In the Zambian communities the predominant negative confounding factors were body mass index and HIV status. The Western Cape communities had the same negative confounding factors, while the predominant positive confounding factor was age.

On combined analysis, there was evidence of a contribution of hyperglycaemia to the population prevalence of tuberculosis throughout the spectrum of hyperglycaemia (Table 4). However, the PAFs of prevalent TB to hyperglycaemia were small, particularly for the higher RBG cut-offs. For RBG concentration ≥11.1 mmol/L the PAF of prevalent TB was 0.99%, 95% CI [0.12–1.85]. When analysed as separate locations, the confidence intervals for the PAFs were wide and showed less evidence for a contribution of hyperglycaemia to the population prevalence of tuberculosis in both Zambia and Western Cape (Table 3).

When stratified by age, for the highest RBG cut-off, the PAF of prevalent TB to hyperglycaemia increased with increasing age in Western Cape, reflecting the higher prevalence of hyperglycaemia in older age groups (Table 5). In Zambia, for this highest RBG cut-off ≥11.1 mmol/L, there remained little evidence for a contribution of hyperglycaemia to the population prevalence of tuberculosis despite the rising prevalence of hyperglycaemia with increasing age, though confidence intervals were wide (Table 5).

**Table 5:** Population attributable fraction of prevalent tuberculosis to hyperglycaemia for Zambian and Western Cape communities, stratified by age, using random blood glucose concentration cut-off 11.1 mmol/L

Age (years)	Zambia		Western Cape	
	Hyperglycaemia prevalence (%)	PAF (95% CI)	Hyperglycaemia prevalence (%)	PAF (95% CI)
18–24	0.42	0.00 (0.00–0.96)	0.27	0.16 (0.00–0.52)
25–29	0.51	0.00 (0.00–1.16)	0.42	0.24 (0.00–0.70)
30–34	1.22	0.00 (0.00–2.72)	0.75	0.43 (0.00–1.05)
35–39	1.18	0.00 (0.00–2.63)	2.04	1.18 (0.07–2.28)
40–49	2.27	0.00 (0.00–4.95)	4.26	2.47 (0.67–4.24)
50–59	4.69	0.00 (0.00–9.88)	7.76	4.50 (1.70–7.22)
60+	4.60	0.00 (0.00–9.70)	8.65	5.01 (1.97–7.97)
Total	1.45	0.00 (0.00–3.21)	2.83	1.64 (0.28–2.99)

*PAF* population attributable fraction, *CI* confidence interval; Hyperglycaemia defined as a random blood glucose concentration ≥11.1 mmol/L; Negative PAFs were given a value of zero

For purposes of comparison to HIV, the PAF of prevalent TB to HIV was 12.72%, 95% CI [7.70–17.47] in the Zambian communities, and 11.72%, 95% CI [8.25–15.06] in the Western Cape communities.

For purposes of comparison to self-reported known diabetes, of individuals with a RBG <11.1 mmol/L, 1.7% in Zambian and 6.8% in Western Cape communities reported having a previous diagnosis of diabetes, and 0.3% and 2.0% respectively reported being on treatment for diabetes. Of individuals with a RBG ≥11.1 mmol/L, 27.9% in Zambian and 57.5% in Western Cape communities reported having a previous diagnosis of diabetes, and 24.0% and 49.1% respectively reported being on treatment for diabetes. Incorporating participants with self-reported previously diagnosed diabetes into the highest category of hyperglycaemia made little difference to the odds ratio point estimates in Zambia and reduced the association seen with RBG concentration ≥11.1 mmol/L in Western Cape towards the null. Defining diabetes by self-report, the odds of prevalent TB for individuals with diabetes compared to those without was 0.83, 95% CI [0.20–3.46] in Zambia and 0.76, 95% CI [0.44–1.32] in Western Cape. For individuals in Western Cape with self-reported diabetes and RBG ≥11.1 mmol/L the odds of prevalent TB was 1.55, 95% CI [0.60–4.01] compared to individuals who did not report diabetes. For those with self-reported diabetes and RBG <11.1 mmol/L this odds ratio was 0.61, 95% CI [0.32–1.18].

Subgroup analyses were not possible for the Zambian data using the highest RBG cut-off ≥11.1 mmol/L due to limited data, and no difference was seen for gender and HIV categories using the RBG cut-off ≥9.0 mmol/L. In the Western Cape using the RBG cut-off ≥11.1 mmol/L, the point estimate of the adjusted odds of hyperglycaemia on prevalent tuberculosis was higher among women than men (for men OR = 1 · 92, 95% CI [0 · 55–6 · 68]; for women OR = 2 · 58, 95% CI [1 · 24–5 · 35]; but there was no evidence the odds ratio differed for men and women, test for interaction *p* = 0 · 68). It was higher among those with HIV than those without HIV (among those with HIV OR = 5 · 34, 95% CI [1 · 56–18 · 23]; among those without HIV OR = 1 · 90, 95% CI [0 · 89–4 · 04]; but the evidence for interaction was weak (*p* = 0 · 17)).

## Discussion

This is the first ever population based study of prevalent tuberculosis diagnosed microbiologically and glycaemia. We used participants who were randomly selected from the community rather than exploring the association among participants who had already been diagnosed with tuberculosis or hyperglycaemia. Among the Zambian participants of our study there was good evidence of a positive linear association between hyperglycaemia and prevalent tuberculosis, and weak evidence for the same association in Western Cape. When data from the two locations were combined, there was evidence of association between hyperglycaemia and prevalent pulmonary tuberculosis across the spectrum of hyperglycaemia. On combined analysis the odds of prevalent tuberculosis was greatest for individuals with the highest level of glycaemia, though the magnitude of association was small. Those with a RBG concentration ≥11.1 mmol/L, had 2.15 times the odds of prevalent tuberculosis than those with a RBG concentration <11.1 mmol/L.

This association seen in the study communities between hyperglycaemia and prevalent tuberculosis is consistent with the association seen elsewhere in the world between diabetes and prevalent TB [17, 18] and also between diabetes and active diagnosed TB [8–10].

Assuming causation, hyperglycaemia contributes little to the prevalence of tuberculosis throughout the spectrum of hyperglycaemia in the Zambian and Western Cape populations. This suggests that hyperglycaemia has only a small impact on the prevalence of TB in the study areas of Zambia and Western Cape despite the positive association seen between hyperglycaemia and prevalent TB in these locations. When stratified by age, however, we can see that the contribution of hyperglycaemia to the prevalence of tuberculosis in Western Cape is greater for older age groups, reflecting the higher prevalence of hyperglycaemia among older individuals. The same trend is seen in the Zambian study population, but the lower prevalence of hyperglycaemia in this setting means that confidence intervals are wide and point estimates remain low even for the oldest age groups.

The combined odds ratios were weighted towards the Western Cape estimates due to the larger number of individuals with hyperglycaemia and tuberculosis in this setting, particularly for RBG concentration ≥11.1 mmol/L. Given the uncertainty of the Zambian odds ratio for this higher level of glycaemia the combined analyses yield the more reliable conclusions. However, regardless of which analysis is used, the conclusion remains the same, that the contribution of hyperglycaemia to the population prevalence of tuberculosis is low.

In this study we measured hyperglycaemia using a single RBG test. To optimise the accuracy of the test research staff were carefully trained and tested, and the point-of-care measure was carefully calibrated using standardised control solution, although not validated against laboratory glucose analyses. Use of this point-of-care test enabled glucose measurement of many participants in a large-scale field study located within the community. Use of a test that is more complicated to administer, such as a fasting blood glucose, oral glucose tolerance test or glycated haemoglobin, would have been logistically challenging and potentially less acceptable to participants, resulting in a much lower uptake of eligible participants and possibly introducing selection bias. Therefore, although not a test to diagnose diabetes, it was felt that the use of a RBG test was most likely to minimise overall bias and loss of study power in this setting of a large-scale population survey, spanning communities and countries. Therefore, rather than diagnosing diabetes, we have measured glycaemia and explored the effect of hyperglycaemia on tuberculosis prevalence. RBG tests normally have good specificity but sub-optimal sensitivity for diabetes [29, 30]. A study in China found measurement of RBG concentration with a cut off of 11 · 1 mmol/L to have a sensitivity for diabetes of only 54 · 8% compared to an oral glucose tolerance test. [31] This would suggest that association seen with hyperglycaemia based on RBG concentration would be an under-estimate of any association with diabetes. However, in the context of active TB disease the specificity of this test for diabetes could also be reduced, as those with stress-induced hyperglycaemia secondary to their TB disease could also have a high RBG concentration. This would result in an over-estimate of the association with TB based on RBG concentration, compared to association with diabetes. A final consideration is a single RBG measurement fails to give data on chronic hyperglycaemia, so we are unable to explore the effect of chronic hyperglycaemia on tuberculosis prevalence from these data. We did explore the effect of participants having previously diagnosed diabetes who may be on treatment and therefore may be normoglycaemic at the time of RBG testing but have longer term hyperglycaemia. The number of these participants were few in both Zambia and Western Cape and when incorporated into the categories of hyperglycaemia did not increase the odds ratio point estimates of the association with prevalent TB in either study location. Regardless of whether the hyperglycaemia measured was due to diabetes, was a consequence of TB disease or was transient from any other cause, the conclusion remains that in our study communities hyperglycaemia contributes little to the population prevalence of tuberculosis.

The substantial losses of evaluable respiratory samples in this study resulted largely from a failure of the positive mycobacterial control to grow in two of the laboratories in Zambia, causing whole batches to be non-evaluable. This has resulted in reduced study power, but is unlikely to have introduced bias to the study results, as the process could not have been associated with the presence or absence of hyperglycaemia in the individuals affected by these missing data. The missing glycaemic data is similarly unlikely to produce bias and was probably the consequence of the lack of prioritisation during data collection, a consequence of nesting this study within a larger cluster-randomised trial. Therefore, this too is unlikely to have been associated with the presence or absence of disease and so is also unlikely to have introduced substantial bias.

The participants in this study were randomly selected from their communities and so are representative of the general population within each community. The communities included were from urban and peri-urban settings and so rural populations are under-represented in this study. The prevalence of both hyperglycaemia and tuberculosis would therefore likely be lower in a general population sample.

In subgroup analysis, the association between hyperglycaemia and prevalent TB among those with HIV was stronger than among those without HIV, which could suggest that hyperglycaemia and HIV work synergistically to increase one’s risk of TB, or could instead reflect an increase in stress-induced hyperglycaemia among those with HIV compared to those without HIV. These findings should be seen as hypothesis generating as the evidence for effect modification was weak and our data are underpowered for formal assessment of effect modification.

## Conclusion

In our study communities in Zambia and Western Cape, there is evidence for a positive linear association between hyperglycaemia and prevalent pulmonary tuberculosis. On combined analysis, individuals with RBG concentration ≥11.1 mmol/L had 2.15 times the odds of prevalent tuberculosis than individuals with a RBG concentration <11.1 mmol/L. Despite this, assuming causation, hyperglycaemia contributes little to the tuberculosis prevalence in our study communities. Investigation of the associations between hyperglycaemia, diabetes and active diagnosed tuberculosis in these study communities would be a valuable addition to the findings from this study, and would allow for sub-group analysis of association with smear-negative, smear-positive and drug-resistant tuberculosis.

## Abbreviations

CI: Confidence interval
FBG: Fasting blood glucose
HbA_1c_: Glycated haemoglobin
HIV: Human immunodeficiency virus
OR: Odds ratio
PAF: Population attributable fraction
RBG: Random blood glucose
TB: Tuberculosis
WC: Western Cape
